# Innate airway immune response to fungal allergens

**DOI:** 10.3389/fimmu.2026.1870612

**Published:** 2026-06-24

**Authors:** Aiswarya Chattuparambil Binoy, Sydney Brack, Taylor A. Doherty

**Affiliations:** 1 Division of Allergy and Immunology, Department of Medicine, University of California, San Diego, La Jolla, CA, United States; 2 Veterans Affairs San Diego Health Care System, La Jolla, CA, United States

**Keywords:** alarmins, *Alternaria*, asthma, chronic rhinosinusitis, fungal allergen, IL-33, innate lymphoid cells, TSLP

## Abstract

Fungi are ubiquitous in our environment, and inhaled exposure of their spores is associated with the development of upper and lower respiratory airway diseases including asthma and chronic rhinosinusitis (CRS). CRS and asthma associated with fungal sensitization also tend to be more severe compared with other endotypes. *Alternaria* and *Aspergillus* species have specifically been recognized as the primary fungal allergens that stimulate a robust innate immune response, potentially leading to allergic sensitization and type 2 inflammation. Other fungi, such as *Cladosporium*, *Penicillium*, and *Candida*, are also associated with airway inflammatory disease. Fungal proteases disrupt airway mucosal barriers and activate protease-activated receptors (PARs), leading to a release of alarmin cytokines, including IL-25, IL-33, and thymic stromal lymphopoietin (TSLP), that drive innate type 2 inflammatory responses. Recent studies have also shown that protease allergens can cleave the protease-sensing domain of IL-33, generating a more active form. Alarmins activate innate immune cells including group 2 innate lymphoid cells (ILC2s), dendritic cells, mast cells, and eosinophils which contribute to epithelial mucus production, airway hyperresponsiveness, and tissue remodeling. This review aims to detail mechanisms of fungal allergen-induced airway inflammation and identify gaps in understanding and therapeutic opportunities.

## 1 Introduction

Multiple studies of allergic populations have shown patterns of reactivity to fungal species, with *Alternaria alternata* (*A. alternata*) and *Aspergillus fumigatus* (*A. fumigatus*) accounting for the main fungal sensitizers (4, 5). Exposure to *Alternaria* has been associated with severe asthma exacerbations, particularly in children (6, 7). Climate and weather patterns are implicated in asthma exacerbations, with thunderstorms increasing the risk ratio of asthma events (8). While the correlation between the climate and asthma events has mainly been attributed to airborne pollen, fungal spore levels also increase after extreme weather events such as thunderstorms (9) and floods (10). Important determinants of airway immune reactivity to fungi include fungal spore size and thermotolerance (11).

While fungal spores are known to be abundant outdoors, multiple studies have demonstrated the presence of fungal allergens in urban indoor settings and in hospital environments via ventilation systems (12–14). *Alternaria alternata*, *Aspergillus versicolor* and *Cladosporium sphaerospermum* have been isolated at high levels indoors, with animal studies demonstrating the induction of both eosinophilic and neutrophilic airway inflammation (15–18). The ubiquity of both indoor and outdoor fungal allergens further emphasizes the importance of research exploring the mechanisms of fungal exposure and disease severity.

The heterogeneity of innate immune responses to fungal pathogens remains insufficiently explored, particularly regarding both inter-species diversity and intra-species heterogeneity. Innate type 2 (T2) inflammation is driven by activated group 2 innate lymphoid cells (ILC2s) that also bridge to that leads to antigen-specific adaptive T-helper (Th2) cell responses. In chronic T2 diseases, both ILC2s and Th2 cells types produce IL-4, IL-5, and IL-13, which promote eosinophilic inflammation, mucus production, hyperresponsiveness, and tissue remodeling. In this review, we highlight mechanisms of innate airway immune responses to fungal allergens that are species or component-dependent, and how that may alter the downstream inflammatory phenotype. Understanding the how initial encounter between the fungal allergen and the host is integral to the development of novel therapeutic strategies that may prevent and treat allergic airway diseases.

## 2 Methods

A literature search was performed using the PubMed database across human studies, animal models, *in vitro* systems, and clinical trials. Original English-language research articles including clinical studies between January 2016 to 2026 were filtered for inclusion. Search keywords using Boolean operators included terms related to innate immunity and fungal allergens, fungal proteases, TLR, PAR, and airway disease. Studies that explored mechanisms of epithelial barrier-fungal allergen contact were included and we prioritized results that focused on type 2 inflammation and airway disease. Studies lacking sufficient methodological detail and non-peer-reviewed publications including conference abstracts editorials were excluded. Additional screening was performed by manually screening through reference lists of highly cited peer-reviewed original research articles.

## 3 Interactions between fungal allergens and epithelial cells

**Figure 1:**
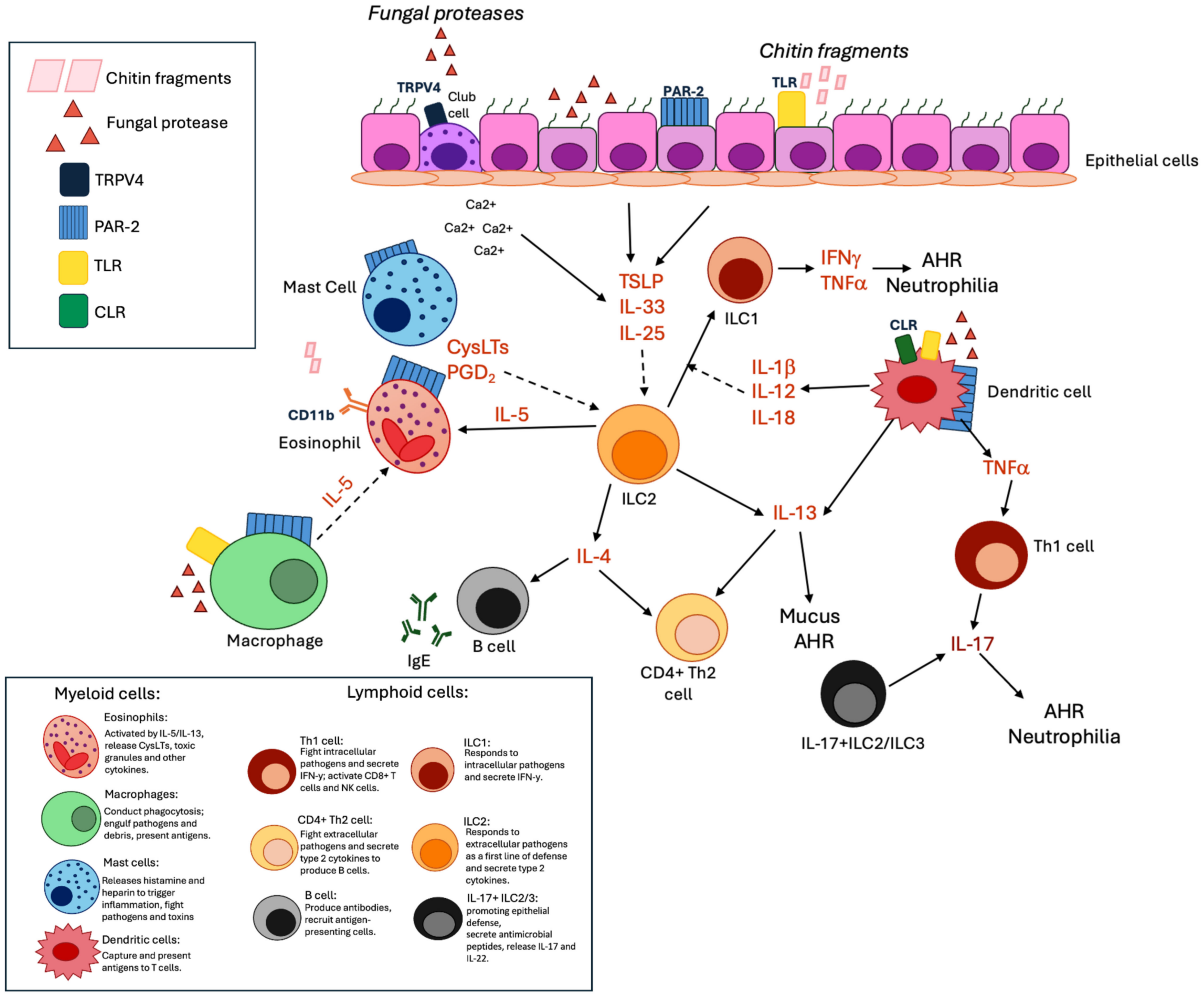
Multiple mechanisms of fungal allergen-driven innate immune airway responses, indicating primary sensors on both epithelial and immune cell surfaces, mediators released upon allergen contact, and effector cell responses to mediator or allergen contact. Fungal proteases activate protease-activated receptors (PARs) and C-type Lectin receptors (CLRs) expressed on epithelial and other immune cell surfaces, while fungal cell wall components such as chitin are recognized by Toll-like receptors (TLRs). These interactions induce the release of alarmins such as thymic stromal lymphopoietin (TSLP), interleukin-33 (IL-33), and interleukin-25 (IL-25). These alarmins consequently activate 2 innate lymphoid cells (ILC2s) that affect a host of other effector immune cells.

Immune responses to fungal spore exposure are first orchestrated by the innate immunity at the mucosal epithelial barriers throughout the respiratory tract (**Figure 1**). Fungal allergen protease activity has been demonstrated to disrupt the airway epithelium. For example, the serine protease activity of *A. alternata* has been shown to initiate ILC2-driven lung inflammation in mice via epithelial barrier disruption and the consequent release of alarmins such as IL-33 (19). *A. alternata* induces the production of reactive oxygen species (ROS) and results in reduced transepithelial resistance (TER) that is abrogated by serine protease inhibitor administration (20). Further, human bronchial epithelial (BEAS-2B) cells, *A. alternata* induced the rapid release of ATP and IL-8 that was dependent on serine, but not cysteine, protease activity (21). Additionally, a role for serine proteases during *A. alternata*-induced ILC2 expansion was demonstrated as secretory leukocyte protease inhibitor (SLPI) could inhibit epithelial release of IL-33 and neutrophil elastase which promotes cleaves IL-33 into a more active form (22, 23).

The induction of airway epithelial IL-8 secretion *in vitro* by *Aspergillus fumigatus* has been shown to be dependent on its protease secretion profile, and influenced by whether the strain is low or high protease-producing (24). Interestingly, administering a cysteine, but not serine, protease inhibitor drastically reduced IL-8 levels, while metalloprotease inhibition modestly reduced IL-8 levels, but led to larger reductions in IL-6. Thus, the fungal-protease specificity determines the type of cytokine release, both across species and within strains of the same species.

Other fungal proteases include aspartyl proteases, which are specifically made by the fungus *Candida albicans*. A recent report demonstrated that chitotriosidase (CHIT1) catalyzes the generation of soluble *C. albicans*-derived chitin oligomers from insoluble chitin, enabling immune recognition by macrophages (25). However, candidal aspartyl proteases Sap2 and Sap6 break down CHIT1 to evade the host immune detection. In another study, Sap6 also bound cell surface integrins and induce epithelial cell apoptosis as part of the pathogen evasion process (26). Overall, fungal proteases represent bona fide allergens with a wide array of pro-inflammatory innate immune effects in the airway (**Table 1**).

**Table 1:** Table summarizing the fungal allergen specific differences and similarities in the innate immune airway response, indicating the fungal components beign sensed, host sensors, mediators released upon allergen contact, and activated immune cells.

Fungal Species	Fungal components sensed	Primary host sensors	Mediators released in response	Activated immune cells
Alternaria alternata	Chitin, serine proteases	PARs, STAT6, TLRs	IL-33, TSLP, CysLTs, ATP, IL-8,	ILC2, Th2 cell polarization, mast cells, DCs, eosinophils
Aspergillus fumigatus	Chitin, serine proteases	PARs, CLRs, TLRs	IL-33, TSLP, IL-17A, NETs, IL-22, IL-12	ILC1, ILC2, mast cells
Candida albicans	Chitin, serine proteases, aspartyl proteases	PARs, GP1bα, TLRs, CLRs	IL-33, TNF-α, IL-6, IL-10, CCL3, CCL4, NETs, DKK1, EETs	Th2 cell polarization, mast cells, Tregs, DCs, macrophages
Cladosporium sphaerospermum	Aspartyl proteases further investigation required	Unknown	Unknown	Unknown
Penicillium chrysogenum	Serine proteases, further investigation required	Unknown	Unknown	Unknown
Aspergillus versicolor	Serine proteases, further investigation required	Unknown	Unknown	DCs, monocytes, ILC2s

### 3.1 Protease-activated receptor sensing of fungal allergens

Protease-activated receptors (PARs) are 7-transmembrane G-protein-coupled receptors found on a myriad of immune and structural airway cells, including epithelial cells. There are four types of PARs: PAR1, PAR2, PAR3, and PAR4, with PAR2 being particularly important in modulating the immune response. PARs were initially discovered as receptors for the serine protease thrombin to bind and activate to initiate the process of coagulation (27), however, they have also been shown to play an important role in fungal allergen-mediated inflammation.

PARs have demonstrated importance in the activation of ILC2s upon fungal allergen contact via the release of thymic stromal lymphopoietin (TSLP) and IL-33 alarmins from epithelial cells. TSLP and IL-33 have also been shown to synergistically augment each other’s protein release after *Alternaria* challenge by increasing the expression of each other’s receptor on mouse lung ILC2s (28) and human cultured ILC2s (29). Knocking out the IL-33 receptor in allergen-treated mice was shown to enhance TSLP production and stimulates IL-9+ and IL-13+ ILC2s, leading to the persistence of type 2 asthma and demonstrating the cooperative abilities of TSLP and IL-33 (30). TSLP, an IL-17-like cytokine, is primarily expressed in epithelial cells and epidermal keratinocytes. TSLP was shown to be induced in BEAS-2B cells by exposure to allergen-derived proteases. Infecting BEAS-2B cells with small interfering RNA for PAR-2 (31) partially blocked the TSLP-inducing activity of *Alternaria*, implicating a role for PAR-2 activation in TSLP induction.

The connection between PAR-2 activation and IL-33 release has been extensively studied. IL-33 is a nuclear cytokine primarily found in epithelial barrier tissue and lymphoid organs. Responsiveness of ILC2s to IL-33 is mediated by the transcription factor Gfi1, which targets and activates *Il1rl1*, the gene that encodes the ligand-binding subunit of the IL-33 receptor (ST2), resulting in the activation of the IL-33/ST2-ILC2 axis to induce allergic inflammation. In the pathophysiology of chronic rhinosinusitis with nasal polyps (CRSwNP), *A. fumigatus* was shown to upregulate IL-33 from human sinonasal epithelial cells (SNECs) and activate ILC2s that produce IL-13, key drivers of eosinophilic inflammation, in a PAR-2 dependent fashion (32).

While previous *in vivo* mouse models indicated that *A. alternata* triggered an innate immune response in a PAR-2 manner, other studies support PAR-2-independent mechanisms. Snelgrove et al. first demonstrated in 2014 that blocking PAR-2 receptor activation by endogenous proteases in the allergen inhibited *A*. alternata-induced IL-33 release into the bronchoalveolar lavage fluid (BALF) by only 68%, indicating the presence of a distinct PAR-2 independent mechanism (33). In a study by Doherty et al., an innate eosinophilic response one day after *Alternaria* exposure was shown to be mediated by STAT6 and independent from PAR-2 activation, with no direct reduction in eosinophilic inflammation in PAR-2–deficient mice (34). Interestingly, the innate eosinophilic lung inflammation was only induced by *Alternaria alternata*, but not by *Aspergillus fumigatus* extract (35), indicating that *A. alternata* uses a unique PAR2-independent pathways to induce rapid type 2 lung inflammation. In contrast, in a model using *Alternaria* filtrate given over 8 days to mice, PAR-2 was required for the development of lung inflammation (19). Differences in the source and processing of *Alternaria*, along with the duration of protocols, may partially explain the varied dependency on PAR-2 in these models. *In vitro* studies with human bronchial epithelial cells demonstrated that *A. alternata* induced a transient ATP release via the VDAC-1 channel (36). The released ATP then binds to the P2Y2 receptor on human bronchial epithelial cells and mouse airways to activate a Ca2+ influx and IL-33 release, in a PAR2-independent manner (37). Whether the activation of this receptor is exclusive to *A. alternata* is yet to be determined. Notably, purified Alp1 protease from *A. fumigatus* can promote AHR and lung eosinophilia independent of PAR-2 (38).

Thus, there appears to be a broad range of epithelial responses dependent on fungal protease detection via PARs but also other components, such as perforation and calcineurin, which trigger other downstream signaling pathways. Which sensing mechanism is activated appears to be dependent on both the fungal species and the component that is being sensed, and further investigation is needed into how complex spores that humans inhale induce these multiple pathways that lead to type 2 inflammation.

### 3.2 PRR sensing of fungal components

Toll-like receptors (TLRs) and C-type lectin receptors (CLRs) are subclasses of pattern recognition receptors (PRRs) (39) that sense conserved pathogen-associated molecular patterns (PAMPs) and damage-associated molecular patterns (DAMPs) from bacteria, viruses, and fungi. They play critical roles in mediating the anti-fungal immune response through sensing of a variety of fungal components.

Chitin (poly(β- (1–4)-N-acetyl-D-glucosamine) is a structural component of the fungal cell wall and is directly sensed by TLR2 (40). In a study conducted by Yan et al. in 2021, TLR2 was shown to play an immunotolerant role by driving gasdermin D (GSDMD)-dependent pyroptosis (41). Interestingly, the cleaved fragment of GSDMD p40 NT-Gsdmd also leads to pore formation in the cell membrane resulting in IL-33 secretion which drives innate type 2 inflammation (42). Jung et al. (43) further demonstrated that chitin-activated ILC2s participate in a feedback circuit by inducing acidic mammalian chitinase (AMCase), which degrades the accumulated chitin and promotes epithelial repair in response to acute epithelial injury. TLR-2 ligand Zymosan A from fungi induced chitinase activity in a human monocyte cell line and airway hyperresponsiveness in mice (44). Thus, TLR2 is linked to both sensing chitin and non-chitin components of fungal cell walls and induce chitinase responses often found in type 2 inflammation.

Beyond chitin, there are other fungal components that induce TLR-mediated immune responses. The major IgE-binding allergen Alt a1 of *Alternaria alternata* directly induces IL-8 and MCP-1 production from epithelial cells and is dependent on both TLR2 and TLR4 (45). Another component of *Alternaria* binds to TLR3 to prevent the induction of mouse bone marrow-derived dendritic cell production of type I interferons that could alter the balance between IFNs/T2 immunity (46). A link between fungal allergen, coagulation, and TLR4 was discovered as fungal protease from *Aspergillus oryzae* cleaved fibrinogen, which acted as a TLR4 ligand to drive type 2 lung inflammation in WT mice, but not TLR4-/- mice (47). *Candida albicans* also has unique ways of inducing TLR-mediated immunity. Extracellular vesicles (EVs) from *C. albicans* interact with TLR4 on murine bone-marrow-derived dendritic cells, leading to IL-6 production (48). Further, a secreted cysteine-rich featured protein (SCP) Sel1 released by *C. albicans* was also induced murine bone-marrow-derived macrophage release of TNF, IL-1β and IL-6 via both TLR2 and TLR4 (49). These studies emphasize both the heterogeneity in TLR-mediated immune responses between fungal species, as well as the variety of specific components within a species that act through TLRs.

### 3.3 Other mechanisms of fungal allergen sensing

Additional components of fungi are detected by the epithelial barrier. A very recent study provided a novel mechanism of how *Alternaria* induces IL-33 and T2 lung inflammation (50). Aeg-S and Aeg-L purified from *A. alternata* extract were required for T2 allergenicity by creating epithelial perforations that trigger IL-33 release and lead to Ca2+ influxes and MAPK pathway activation. *A. alternata* strains devoid of *Aegs* and *aegl* failed to induce IL-33 *in vitro* and *in vivo*, along with reduced epithelial MEK1/2 phosphorylation (50). Additional pathways may also be used by *A. fumigatus* to induce IL-33 release and lung inflammation. *A. fumigatus* signaled through epithelial TRPV1 and TRPV4, leading to Ca2+ release via calcineurin which was essential for IL-33 release and ILC2 activation (51, 52). Type 2 responses to *Candida albicans* may also involve platelets representing a novel mechanism apart from epithelial activation. The peptide candidalysin activated platelets via the GP1bα receptor to produce the Wnt antagonist Dickkopf-1 (DKK1) which led to enhanced Th2 cell-driven lung inflammation in mice (53).

Interesting, not all fungal components are pro-inflammatory in nature. A recent report demonstrated that melanin derived from the *A. fumigatus* cell wall can block calcium signaling in human airway epithelial cells which suppresses the secretion of chemokines CXCL1 and CXCL8 (IL-8) and prevents neutrophil recruitment (54). *A. fumigatus* melanin thus appears to have immunoregulatory effects, which may counteract the *A. fumigatus*-derived proteases that increase IL-8. These studies further highlight the variation in epithelial cell responses to different fungal components.

## 4 Innate immune cell responses to fungal allergens

### 4.1 ILC2s

ILC2s are one subset of innate lymphoid cells (ILCs) that also include ILC1s and ILC3s (55–58). ILC cytokine profiles resemble conventional CD4^+^ Th1, Th2, and Th17 subsets, but unlike T cells, ILCs are not antigen specific. ILCs originate as common lymphoid progenitors (CLPs) and require transcription factors, including inhibitor of DNA binding 2 (Id2), thymocyte selection-associated high-mobility group box protein (TOX), nuclear factor interleukin-3 regulated (NFIL3), and signaling through γ chain (γc) and Notch pathways. ILC1s promote responses to viral infections, intracellular pathogens, and tumors, producing cytokines IFN-γ and TNF-α. Natural Killer cells (NK) are often classified in group 1 ILCs given their similar cytokine and transcription factor profiles to lineage-negative ILC1s. ILC2s are regulated by the master T2 transcription factor GATA-3 and produce IL-4, IL-5 and IL-13, and the growth factor amphiregulin. ILC2s play key roles in immune responses to helminths, allergens, and some viral infections, in addition to roles in tissue repair. ILC3s produce IL-17 and IL-22, leading to neutrophilic responses against extracellular bacteria and fungi.

ILC2 activation is integral to the pathophysiology of fungal-mediated T2 airway immune responses (**Figure 1**). ILC2s are highly activated epithelial-derived alarmins IL-33, IL-25, and thymic stromal lymphopoietin (TSLP). Fungal allergens, including *Alternaria alternata*, rapidly induce high levels of IL-33 levels in mice that then activate lung ILC2s to produce type 2 cytokines (59–61). Further, *Alternaria* also induces TSLP *in vitro* when cultured with human lung epithelial cells (31). The ILC2 response in mice to *Alternaria* airway exposure is so robust that most studies to understand novel mechanisms of ILC2s utilized variations of the model, thus highlighting the importance of the ILC2 response to *Alternaria*. The role of ILC2s to other fungal allergens is not as well documented, and early studies showed that Candida and Aspergillus do not induce the same level of IL-33-driven ILC2 activation as *Alternaria* (34, 61). However, invariant NKT cells can directly produce type 2 cytokines in response to Aspergillus glycolipids and induce AHR in mice (62).

In addition to rapid T2 cytokine producers, ILC2s also bridge innate to adaptive T2 immune responses. One report demonstrated that OX40L expression by ILC2s is required for IL-33-driven Th2 and Treg cell expansion in an *Alternaria alternata model*, indicating that ILC2s may control the T2 adaptive responses to fungal allergen (63). Very recently, ILC2s were also found to be a critical source of GM-CSF production in response to Alternaria exposure in mice that led to increased activated dendritic cell migration to the lymph nodes and subsequent CD4+ Th2 cell expansion (64). In mice specifically lacking ILC2s, the DC migration and Th2 cell responses did not occur when exposed *to Alternaria*.

In addition to promoting Th2 cell responses, IL-5 production by ILC2s in response to an *A. alternata* challenge is essential in supporting murine B1 cells, where the conditional deletion of *Il5* in ILC2s resulted in defective B1 cell development and immunoglobulin production (65), thus highlighting the multi-pronged role of ILC2s in driving adaptive immunity in response to fungal allergen exposure.

The role of conventional ILC3s in fungal allergen exposures is not clear. However, ILC subsets display significant plasticity and *Alternaria* exposure has been shown to lead to hybrid IL-17-producing ILC2s that have features of both ILC2s and ILC3s (66). These hybrid populations have also been detected in severe human asthma sputum (67). While further research is needed to determine the precise mechanisms by which fungal allergens may cause ILC3-mediated IL-17A release, this emphasizes the far-reaching effects of fungal pathogens in inducing both type 2 and non-type 2 inflammatory responses.

Though ILC1s are mainly involved in the antiviral and anti-tumor responses, they might be indirectly activated by certain fungal allergens. Recognition of *A. fumigatus* by C-type lectin receptors (CLRs) on dendritic cells was demonstrated to release a host of pro-inflammatory cytokines such as IL-12, which has been shown to drive ILC2s to differentiate into T-bet expressing and IFN-γ producing ILC1s in a murine model (68). Consistent with other ILC plasticity studies, Cavagnero et al. (69) demonstrated that stimulation of STING during *Alternaria* exposure induced a shift from ILC2s to ILC1s that was dependent on the type 1 IFN receptor. Other studies have found ILC1-like populations or “ex-ILC2s” that behave more like ILC1s after stimulation of type 1 cytokines such as IL-12 (70, 71). However, any potential direct effect of fungal-allergen exposure on ILC1s remains to be studied and will also require further modeling in human studies.

### 4.2 Dendritic cells

Dendritic cells (DCs) are critical bridges between innate and adaptive immunity. With recent characterization of DC subtypes, strides have been made in understanding the various responses in regulating type 2 inflammation in response to fungal allergens. Cook et al. recently illustrated that a Mgl2^+^ CD301b^+^ conventional DC2 population was responsible for driving Th2 fungal inflammation, but not Th17, while other DC clusters were not essential for driving type 2 inflammation (72). Interestingly, *A. fumigatus* model failed to induce IL-33 and TSLP, like previous *A. alternata* models, indicating that *A. fumigatus* might lead to unique DC-driven responses more so than alarmin responses. Type 1 conventional DCs (cDC1s) have also been shown to act as negative regulators of type-17 immunity during fungal lung infections, further emphasizing the diversity in the role of dendritic cells in the immune response to fungi (73). As humans with fungal type 2 responses largely inhale spores, the form of the inhaled fungi may be critical to how DCs are activated. Houlder et al. demonstrated that resting conidia do not activate DCs, but swollen spores after 3 hours strongly triggered a DC response (74). Thus, the response to various fungi by DCs is not uniform, and further understanding is needed to elucidate the role of specific fungal species and forms that take part in driving DC-mediated Th1/Th17/Th2 crosstalk.

### 4.3 Macrophages

Macrophages are a heterogeneous cell population, polarizing towards either M1 or M2 phenotypes, that are pro-inflammatory or anti-inflammatory, respectively. As phagocytes, they recognize and respond to invasive fungi by through TLRs and CLRs. The M1 phenotype contributes to IFNγ-mediated responses and M2 macrophages are induced through IL-4/13-STAT6 signaling (75). The M1 phenotype is associated with fungal clearance, and M1 polarization is positively regulated by STAT1 (76). *A. fumigatus* ingestion by macrophages has been shown to trigger mitoROS via reverse electron transport upon exposure to swollen conidia, resulting in elevated IL-1β expression (77), a hallmark of M1 macrophages. Another study demonstrated that M1-macrophage-derived Galectin-9 secreted upon conidia interaction was also responsible for activating NK cells and regulating the inflammatory response.

As M2 macrophages display a more immune suppressive phenotype compared with M1 macrophages, further research is required to elucidate what factors maintain the prevention of M1-M2 polarization during *A. fumigatus* infection, ensuring effective clearance. Interestingly, macrophages appear less effective in clearing *A. alternata* as the mycotoxin alternariol suppresses LPS-induced inflammation in THP-1-derived macrophages and induces IL-10, characteristic of the M2 phenotype (78). Interestingly, *A. Alternaria* enhances the loss of alveolar macrophages during influenza infection, compared to only influenza challenges (79). *C. Albicans* hyphae are also adept at immune evasion, exiting macrophages using the pore-forming proteins candidalysin and Gasdermin-D to permeabilize through macrophage membranes, suggesting fungi-specific responses by macrophage populations (80, 81).

### 4.4 Eosinophils and neutrophils

Though eosinophils and neutrophils are often considered downstream effector granulocytes during fungal allergen-induced airway inflammation, they also directly respond to fungal elements. Zaffran et al. demonstrated that the CD48 on eosinophils is an important surface receptor for *C. albicans* recognition via agglutinin-like sequence 6 (Als6) expression *in vitro* and *in vivo* in mice (82). Activated human eosinophil extracellular traps (EETs) are structures released by eosinophils that can entrap fungi. However, in uncontrolled airway disease, EETs accumulate and can promote further airway inflammation. EET release and consequent suppression of fungal metabolism occur in response to *A. fumigatus* and *C. albicans*, but not *A. alternaria* (83–85), emphasizing the diversity of the innate immune response in response to various fungal organisms. The mechanistic reasons for this difference in EET release are yet to be fully characterized but may be important in distinct asthma and CRS endotypes.

Similar to EETs, human neutrophils also release extracellular traps (NETs) upon PRR activation. During NETosis, chromatin de-condensation releases NETs that trap fungal pathogens and can increase the proinflammatory response. The effect of fungal allergens on NETs is not well established, and much of the literature comes from fungal infection models (86). *C. albicans* has been implicated in inducing the release of NETs to capture the yeast and delay hyphal formation (87). However, *C. albicans* can counter this neutrophil response by using the aspartyl protease Sap6 as a ‘trojan horse’, as Sap6 binds integrins on epithelial A549 cells, leading to internalization and evasion (26). In human neutrophils, Sap6 degrades reactive oxygen species–generating NADPH oxidase, thus inhibiting NET production and promoting neutrophil apoptosis. Consequently, at high concentrations of Sap6 and fungal burden, NETosis is impaired, limiting the effectiveness of the neutrophil-mediated immune response. The role of NETs in fungal infections may be fungi-specific. In a mouse model of invasive pulmonary aspergillosis caused by *Aspergillus fumigatus*, PAD4-dependent NET formation impaired fungal clearance, as PAD4-deficient mice exhibited reduced fungal burden and decreased lung injury (88). Thus, NET formation can play either a protective, fungus-clearing role or fuel an exacerbation of tissue damage, depending on fungal interactions, and studies into fungal allergens and NETs are lacking. Though direct neutrophil interactions with fungal allergens are not well described, neutrophils can play an important indirect role in fungal allergen T2 responses through the activation of mature IL-33 through elastase and cathepsin G (23).

### 4.5 Mast cells

Mast cells (MCs) are key effector cells of acute allergic hypersensitivities and chronic T2 inflammatory diseases. MCs are known to contribute to the pathobiology of airway diseases, including asthma and chronic rhinosinusitis with nasal polyps (CRSwNP). As tissue-resident cells, they are found near epithelial barrier sites in the respiratory tract and can thus directly interact with fungal pathogens. MCs can recognize candidalysin from *C. albicans* via CLR Dectin-1, leading to degranulation (89, 90), followed by an increase in cytokines and chemokines, including TNF-α, IL-6, IL-10, and CCL4. Murine MCs have also been shown to exhibit IgE-dependent MC degranulation in response to direct contact with *A. fumigatus* conidia, resulting in bronchial hyperreactivity and an increase in neutrophils in the BAL fluid (91).

Additionally, mast cell crosstalk with ILC2s can significantly enhance the T2 inflammatory response. In an *Alternaria* murine model of lung inflammation, MC proteases have been shown to cleave the central domain of the IL-33 alarmin, leading to enhanced ILC2 expansion (92). IL-9 is also produced by ILC2s and promotes mast cell survival and activation (93). Mast cells are an important source of cysteinyl leukotrienes and Prostaglandin D, which both promote chemotaxis and activation of ILC2s (94–96). Mast cells in the skin have also been co-localized with ILC2s, but studies in the airways of humans are lacking (97). Overall, the crosstalk between ILC2s and mast cells likely supports positive feedback loops to maintain T2 allergic inflammation in response to fungal allergens.

## 5 Therapeutic strategies targeting fungal T2 immunity

The widespread use of biologics in the treatment of severe T2 inflammation in asthma and CRS has been a profound asset in the management of these patients. Conventional treatment of severe T2 airway diseases with excessively high-dose corticosteroids is fraught with side effects and additionally may promote A. fumigatus growth via M1-M2 macrophage polarization and immune suppression (98). There are currently six approved monoclonal antibodies (mAbs) against various T2 targets, including omalizumab, mepolizumab, reslizumab, benralizumab, tezepelumab, and dupilumab. Omalizumab, mepolizumab, and dupilumab are approved in the US for CRSwNP and all have been approved for uncontrolled chronic T2 asthma. Omalizumab is an anti-IgE mAb and has shown some efficacy in severe T2 endotypes of airway diseases that include allergic fungal rhinosinusitis (AFRS) (99) and bronchopulmonary aspergillosis (ABPA) (100). Mepolizumab is an anti-IL-5 mAb with some reported success in treating ABPA (101). Benralizumab is an anti-interleukin-5 receptor α (IL-5Rα) that directly leads to eosinophil apoptosis has also shown some efficacy in ABPA (102) patients. Dupilumab is an anti-IL-4Ra therapy that targets both IL-4 and IL-13 signaling and has been effective in some patients with ABPA (103) and AFRS (104), with additional efficacy against *A. alternata* sensitization when combined with allergen immunotherapy (105).

Tezepelumab, targeting the alarmin TSLP, has had some success in treating ABPA (106) and holds potential in targeting other fungal-mediated airway diseases due to its upstream action that may also reduce ILC2-driven responses. Aside from tezepelumab, there are currently no approved therapies specifically targeting alarmins in T2 airway disease. However, biologics targeting IL-33 and IL-25 are under investigation. In a phase 2 trial, the anti-IL-33 monoclonal antibody Itepekimab (anti-IL-33) improved control in moderate-to-severe asthmatics (107). Tozorakimab is another anti-IL-33 biologic in phase III clinical trials and has demonstrated a successful reduction in inflammation independent of blood eosinophil levels and smoking history in patients with chronic obstructive pulmonary disease (COPD) (108). Astegolimab is an anti-ST2 (IL-33R) monoclonal antibody has been assessed in a phase 2 study in severe asthmatics and showed reductions in asthma exacerbation rates that were similar regardless of eosinophil counts (109). While the anti-IL-25 monoclonal antibody LNR125 has been investigated in its ability to block virus-exacerbated asthma responses, it has not been studied within the context of fungal-exacerbated asthma, emphasizing the need for further exploration of its uses (110).

Given the importance of proteases in fungal allergen responses, PAR-2 antagonists are currently being studied as potential treatments. Despite structural considerations and off-target concerns of PAR-2 antagonist development, there have been some recent insights that hold potential as an upstream target against fungal-mediated airway disease. PAR2 antagonists have reduced airway hyperresponsiveness, airway inflammation, and mucus overproduction in an *A. alternata*-challenged *in vivo* murine model (111, 112). PAR-2 antagonists have yet to be tested in clinical trials, and their translational efficacy in treating asthma and other fungal-mediated airway diseases is not known.

## 6 Conclusions

Climate change and urbanization on the rise and exposure to fungal allergens poses a global health challenge in our indoor and outdoor environments. Patients with airborne fungal allergy have greater severity of disease, and certain fungal allergens, such as *Alternaria alternata*, are associated with near-fatal asthma events. While there has been significant focus on the adaptive immunity of allergen responses, the innate immune system, including epithelial barriers, ILCs, DCs, and mast cells, represents the first lines of defense and bridge to T and B cell responses. Importantly, type 2 lung inflammation from fungal allergens can also completely bypass adaptive immunity and potentially lead to worsening disease. Impediments to the development of specific fungal-allergen targeted therapeutics are due to the heterogeneity of host immune responses and variability in responses to individual fungal components, even within the same species. Though current biologic therapies that largely target type 2 immunity are effective for many, those with refractory fungal allergen-associated airway disease may benefit from novel targeted therapies developed from a deeper understanding of specific fungal allergen mechanisms.
